# Argument Structure and the Representation of Abstract Semantics

**DOI:** 10.1371/journal.pone.0104645

**Published:** 2014-08-11

**Authors:** Javier Rodríguez-Ferreiro, Llorenç Andreu, Mònica Sanz-Torrent

**Affiliations:** 1 Departament de Psicologia Bàsica, Universitat de Barcelona, Barcelona, Spain; 2 Institute for Brain, Cognition and Behaviour (IR3C), Universitat de Barcelona, Barcelona, Spain; 3 Cognitive Neuroscience and Information Technologies Research Program, IN3, Universitat Oberta de Catalunya, Barcelona, Spain; University of Akron, United States of America

## Abstract

According to the dual coding theory, differences in the ease of retrieval between concrete and abstract words are related to the exclusive dependence of abstract semantics on linguistic information. Argument structure can be considered a measure of the complexity of the linguistic contexts that accompany a verb. If the retrieval of abstract verbs relies more on the linguistic codes they are associated to, we could expect a larger effect of argument structure for the processing of abstract verbs. In this study, sets of length- and frequency-matched verbs including 40 intransitive verbs, 40 transitive verbs taking simple complements, and 40 transitive verbs taking sentential complements were presented in separate lexical and grammatical decision tasks. Half of the verbs were concrete and half were abstract. Similar results were obtained in the two tasks, with significant effects of imageability and transitivity. However, the interaction between these two variables was not significant. These results conflict with hypotheses assuming a stronger reliance of abstract semantics on linguistic codes. In contrast, our data are in line with theories that link the ease of retrieval with availability and robustness of semantic information.

## Introduction

Adults produce longer reaction times and are more error-prone when presented with tasks that involve the processing of abstract, compared to concrete, nouns [1]–[4]. Patients with acquired language disorders due to focal brain damage, as well as dementia patients, have also been studied regarding this issue. Thus, whereas semantic dementia patients present a relative preservation of abstract concepts [5], [6], a significant amount of aphasic [7]–[10] and dyslexic [11] patients show a relative impairment of abstract words processing.

Several hypotheses have been proposed to explain the differences between concrete and abstract semantics. The context availability theory [12], [13] suggests that abstract and concrete concepts are represented in a single amodal network of abstract symbols or propositions. This theory ascribes the relative ease in the processing of concrete nouns to the greater richness of their meanings, and the availability of more contextual information in semantic memory supporting their processing. Some studies confirming the role of context availability in concrete and abstract word processing support this view [14]–[16].

The dual coding theory [17]–[19], on the other hand, proposes a different perspective. According to this interpretation, concrete and abstract knowledge is represented in two qualitatively different ways. Sensory and verbal information is processed through different channels, and leads to separate representations: image-based codes for sensory information, and word-based codes for verbal information. Due to their dependence on linguistic codes, the retrieval of information related to abstract concepts has its neural counterpart in brain regions located in the dominant hemisphere. The processing of concrete nouns, however, also involves the activation of other brain regions in either hemisphere, due to their reliance on sensory information. Some neuroimaging studies have provided support for the dual coding theory [20], [21]. Nevertheless, results in conflict with this hypothesis have also been observed [22]–[26].

The lack of agreement regarding how we represent the meaning of nouns referring to abstract entities extends to the verb/action domain. The lexical-semantic representation of concrete verbs has been largely studied, pointing out that the processing of motion verbs involves activity in neuronal networks including sensory and motor regions [27], [28]. Nevertheless, the few studies that have explored the way the meaning of abstract verbs is represented have obtained conflicting results [24], [29]–[31].

Thus, Rüschemeyer, Brass and Friederici [31] did not find differences between the neural correlates associated to the processing of concrete and abstract verb. In contrast, Rodríguez-Ferreiro, Gennari, Davies and Cuetos [30] found specific patterns of neural activity associated to those two verb categories. Abstract verbs elicited stronger activity in frontal regions related to effortful semantic retrieval. Hence, the authors concluded that the disparity between concrete and abstract verb processing is not related to a differential dependence on verbal codes, but with the greater effort on semantic retrieval or semantic property integration required by abstract semantics. Perani et al. [29] also found specific activation patterns for abstract words in frontal regions. However, the authors associated the specific activity in response to verb processing to the automatic retrieval of syntactic information. According to another study by Grossman, et al. [24], this aspect could be a key point in the study of the representation of abstract verbs. These authors ascribed the differential neural activation associated to abstract verbs to the retrieval of the complex network of propositional features that provide abstract verbs with a linguistic context.

The aim of this paper is to analyze the way the meaning of abstract verbs is represented. Given the potential importance of the semantic complexity and contextual information in the retrieval of abstract knowledge, we use verbs with different argument structure and verbs with different argument complexity.

Argument structure, within linguistic theory, is a construct that specifies the relation between the semantics of a verb and its syntactic expression [32]–[34]. The argument structure of a verb specifies combinatory semantic information: the number and type of possible semantic arguments, often referred to as thematic roles; combinatory syntactic information: how these arguments should be expressed syntactically; and non-combinatory semantic information: detailed knowledge about the nature and the frequency of the lexical items that could play the different thematic roles associated to the verb.

Each verb specifies a particular number of arguments, and determines which of these arguments must be obligatorily expressed. For example, the verb “shoot” must have two arguments (transitive verb). In the sentence “The hunter shot the rabbit”, the agent is the hunter who executes the action and the patient is the rabbit who is affected by the action. In contrast, the verb “run” takes only the agent of the action (i.e. who runs) as an argument (intransitive verb). Thus, transitive verbs present a wider argument structure, with more arguments, than intransitive ones.

Moreover, verbs can also differ in syntactic argument complexity. Thus, some verbs allow sentential clauses as complements, making the picture even more complicated. A verb like “explain” allows complex sentential complements in the form of that-clauses (e.g. “he explained that the film was very good”) as well as interrogative (e.g. “he explained why the film was so good”) or exclamation (e.g. “he explained what a good film it was!”) clauses.

Hence, argument structure provides a measure of the intricacy of the linguistic contexts associated to a verb. The amount of arguments a verb allows reflect their semantic-syntactic complexity. On the other hand, nominal or sentential arguments reflect the complexity of contextual information associated with a verb.

Effects of argument structure width on language processing have been reported on language-impaired population. Linguistic production of Broca’s aphasics becomes more difficult as the amount of arguments entailed by the verb increases [35]–[37]. Furthermore, the complexity of the complements, simple nominal or propositional clauses, affects language production of these patients too [36]. Effects of the number of arguments on language processing are also present in developmental language disorders, like Specific Language Impairment [38], [39]. Nevertheless, the opposite pattern, better performance with verbs taking more arguments, has been associated to fluent aphasia [40] and Parkinson’s disease [41]. Finally, effects of argument structure have also been observed on processing speed of healthy volunteers. With some exceptions [42], previous studies have reported a facilitation effect for verbs with wider argument structures in lexical decision tasks [43], [44], what has been interpreted as evidence of ease on semantic integration [43].

Our study is, thus, based on the following hypothesis: if the representation of abstract semantics depends more on the linguistic contexts abstract words are associated to, then greater effects of argument structure, either structure width or argument complexity, should be expected on the processing of abstract compared to concrete verbs. We submit the results of two experiments in which healthy Spanish-speaking participants were presented with verb stimuli varying in imageability and argument structure characteristics. For the first experiment, a lexical decision task with verbs and verb-like pseudowords was constructed. There is evidence that lexical decision is sensitive to semantic effects [45]–[47]. More specifically, imageability effects have repeatedly been reported in this task [48]–[50]. As we have already mentioned, effects of argument structure on lexical decision have also been observed [51]. However, there is still a debate about the sensitivity of lexical decision to detect syntactic-grammatical effects [51], [52]. In order to ensure a deeper level of linguistic processing, a grammatical decision task was designed for a second experiment. We assume that instructions to decide whether a presented word is a verb or not would focus the process to the semantic-syntactic properties of the stimuli and, thus, facilitate the arousal of the sought effects.

## Experiment 1

### Methods

#### Ethics Statement

The study was approved by the ethics commission of the University (Comissió de Bioètica de la Universitat de Barcelona). Written informed consent was obtained from all the volunteers prior to their participation in the experiment.

#### Participants

A group of 39 students took part in the experiment. They were all right-handed native Spanish speakers studying at the University of Barcelona with normal or corrected-to normal vision. They participated in the experiment in exchange of course credits.

#### Materials

Three sets of 40 Spanish verbs each: intransitive verbs, simple transitive verbs and sentential transitive verbs were selected to be used in the study. Argument structure was determined by a search in a syntactic database of Spanish [53]. Verbs that appeared without any complement more than 85% of the times in the database were considered intransitive (e.g. “flotar”→ to float). Verbs that appeared with a simple direct object more than 80% of the times were considered simple transitive (e.g. “atrapar” → to catch). The sentential transitive (e.g. “deducir” → to deduce) category consisted of transitive verbs that were able to take sentential complements, including that-clauses, interrogatives and exclamations. A full list of stimuli and their percentages of appearance with the different argument structures in the syntactic corpus is presented in Appendix S1. Half of the verbs in each group were concrete and half were abstract. The verbs were split into these two categories on the basis of imageability ratings in the LEXESP database [54] through the B-Pal software [55]. A survey with the same characteristics of that used in the LEXESP study (1–7 Likert-like scale, 7 indicating very easily imageable) was conducted in order to get imageability values that were not present in the database. A group of 25 participants different to those that took part in the experimental tasks responded to the survey. A set of 25 items that already appear in LEXESP were also included to get a measure of the comparability of the two studies. No significant differences appeared between our values and those gathered in LEXESP for these items. We provide the results of this survey in Appendix S2. High and low imageability verb sets significantly differed in this value, but were matched on letter and syllable length as well as orthographic neighbourhood size [55] and oral lemma-based frequency counts gathered from the EsPal database [56]. The groups of intransitive, simple transitive and sentential transitive verbs were also matched with each other on these variables. Although high and low imageability subgroups within each transitivity group were matched on lemma frequency, differences in this variable between transitivity groups appeared on the comparison between the low imageability- intransitive and low imageability-simple transitive categories, and the low imageability-intransitive and high imageability-sentential transitive categories. A summary of the characteristics of the experimental stimuli is presented in table 1. Finally, 120 pseudo-verbs were created to be used as filler stimuli (see Appendix S3). The filler list was matched with the experimental list on letter and syllable length. A similar distribution of -ar, -er, and -ir endings (the three possible endings for an infinitive form verb in Spanish) was present in the experimental and filler lists of stimuli. Six practice items were also selected.

**Table 1:** Summary of stimuli characteristics, mean (SD).

Imageability	Imag.	Int.%	Sm Tr.%	St Tr.%	LexicalFrequency.	Syllables	Letters	OrhtographicNeighbourhood
Intransitive								
Low	2.79(0.4)	98.92(3.03)	0	0	12.71(37.93)	2.8(0.52)	6.95(1.1)	1.1(1.74)
High	4.72(0.63)	96.43(5.53)	0.81(3.2)	0	48.16(155.5)	2.65(0.67)	7.05(1.15)	1.2(1.64)
SimpleTransitive								
Low	2.81(0.4)	4.22(4.51)	91.42(4.83)	0.45(1.47)	22.61(34.24)	3(0.46)	7.2(0.83)	1(1.45)
High	4.76(0.79)	5.18(4.61)	91.64(5.2)	0	44.96(47.59)	2.7(0.57)	7.05(1)	1.2(1.91)
SententialTransitive								
Low	2.62(0.44)	13.4(12.64)	75.07(19.4)	33.92(13.15)	26.59(44.57)	2.9(0.45)	7.1(0.91)	0.65(1.18)
High	4.13(0.81)	12.29(11.69)	75.7(20.05)	39.64(24.26)	41.42(45.04)	2.95(0.39)	7.15(0.99)	1(1.62)

%indicates de values are percentage of appearances with that specific argument structure in the corpus.

#### Procedure

Stimuli were presented and reaction times were recorded with the DMDX application [57]. An experimental trial was as follows: first an asterisk was presented as fixation point for 500 ms. Then the stimulus appeared on-screen for other 500 ms. Finally a blank screen was presented for 1500 ms. Participants were instructed to rapidly and accurately press a key with their right hand when the stimulus was a real verb, or a different key with their left hand when it was not a real verb. Stimuli were presented visually with upper-case black letters (Arial font 14 pt) on a light grey screen. The words were written without accent marks because their presence would directly indicate that the word is not a verb (verbs in citation form never carry an accent mark in Spanish). Absence of accent marks is common in written Spanish when upper-case fonts are used. When asked about this matter after the experiment had finished, none of the participants reported any concern regarding the lack of accent marks. The order of presentation of the stimuli, experimental and filler lists was randomized for each participant. Six practice items were presented at the beginning of the experiment. A rest period was introduced after the first 120 stimuli had been presented. After the experiment had finished the participant was debriefed.

### Results

A summary of the latencies and percentages of errors of the participants in each condition is provided in table 2. Data resulting from this experiment are available on demand to the corresponding author. Log (base 10) transformation of the participants’ reaction times, time elapsed between the onset of the target stimulus and the response, was carried out prior to their inclusion in the analyses. Data were analyzed by means of generalized linear mixed-effects modelling with the lme4 package [58] in R [59]. Mixed-effects models let us estimate fixed replicable effects like those of imageability or transitivity, as well as random effects such as unexplained effects due to random variation between items or participants. In order to account for possible effects of stimuli characteristics we introduced letter length and lexical frequency variables as covaraties in the analyses. Following Barr, Levy, Scheepers and Tily [60] we used the maximal random effects structure justified by the design, which included intercepts for participants and items as well as by-participant slopes for the interaction between imageability and transitivity. Using this approach, one compares a model containing the fixed-effects structures of interest with a model that is identical to it but does not contain the fixed effect in question, by means of likelihood ratio tests. Our analyses showed model 1, including the independent variable imageability as well as the two covariates to be more informative than model 0, which included only the two covariates (χ^2^(1) = 14.767, p<.001). Model 2, including both transitivity and imageability as well as the two covariates, appeared to be more informative than model 1 (χ^2^(2) = 19.339, p<.001). The comparison of model 2 with the full model 3, also including the interaction between the two independent variables, yielded no significant results (χ^2^(2) = 0.597, p = .74) indicating that the interaction effect does not improve the model and can, thus, be excluded. Characteristics of the final model (model 2) are presented in table 3. Finally, we also conducted a bayesian analysis by means of the Bayes Factor package [61] in R. The comparison between the full model including the main effects and its interaction and the model including only the main effects yielded a Bayes factor of 0.005. This indicates extreme evidence [62], [63] that our data are more likely to occur under the simpler no-interaction model than under the full model.

**Table 2:** Summary of reaction times, mean (SD), and percentages of errors in the different conditions for the two experiments.

	Imageability	
Lexical Decision	High	Low
Intransitive	742(226) 15.5%	801(261) 15.5%
Simple Transitive	697(202) 1.8%	749(227) 4.3%
Sentential Transitive	690(196) 1%	745(221) 6.1%
	**Imageability**	
**Grammatical Decision**	**High**	**Low**
Intransitive	921(305) 15.8%	988(338) 26.5%
Simple Transitive	843(256) 6.4%	923(316) 8.5%
Sentential Transitive	829(256) 4.6%	899(292) 9.2%

**Table 3:** Summary of models including the two covariates, the two independent variables and their interaction.

	Lexical Decision		Grammatical Decision	
	Estimate	t value	Estimate	t value
(Intercept)	2.772	112.57	2.905	106.30
Imageability				
High vs. Low	0.03	2.86	0.028	2.41
Transitivity				
Intransitive. vs. Simple Transitive	–0.025	–2.49	–0.038	–3.39
Intransitive. vs. Sentential Transitive	–0.034	–3.15	–0.049	–4.37
Frequency	–0.0002	–4.44	–0.0002	–4.52
Length	0.0131	4.36	0.008	2.27
Interactions				
High vs. Low×Int. vs. Simp. Trans.	–0.008	–0.59	0.002	–0.12
High vs. Low×Int. vs. Sent. Trans.	0.002	0.12	0.006	0.41

Significant effects of both imageability and transitivity were observed. High imageability words were found to evoke faster reaction times than those with low imageability values. Planned contrasts (Tukey’s HSD) revealed that intransitive verbs evoked significantly longer reaction times than both simple and sentential transitive verbs (ps<.001). Nevertheless, no significant differences appeared between the two transitive verb subcategories (p = .75).

## Experiment 2

### Methods

#### Participants

Thirty-nine new volunteers with the same characteristics as those in experiment 1 took part in the experiment.

#### Materials

The same experimental stimuli, verbs varying in imageability and argument structure characteristics were used in this experiment. A total of 120 real Spanish nouns and adjectives ending in -ar, -er or -ir were selected to be used as fillers in a grammatical decision task (see Appendix S3). Non-verb stimuli were matched with the verb list on letter and syllable length as well as lexical frequency. A similar distribution of -ar, -er, and -ir endings was present in the experimental and filler lists of stimuli. Six practice items were also selected.

#### Procedure

The grammatical decision task had the same procedure as the lexical decision task in experiment 1, only the filler stimuli list and the initial practice items, changed between them. Participants were instructed to press one key with their right hand when a verb was presented and the other key with their left hand when the presented stimulus was not a verb.

### Results

The same analytic approach used in experiment 1 was applied to the data gathered in this experiment. A summary of the latencies and error rates of the participants is presented in table 2. Data resulting from this experiment are available on demand to the corresponding author. Results were very similar to those observed with lexical decision in the previous experiment. Model 1, comprising the independent variable imageability and the two covariates, appeared to be more informative than model 0, with only the two covariates (χ^2^(1) = 11.013, p<.001). Again, the best model (see table 3) was model 2, which included imageability and transitivity as well as the two covariates (χ^2^(2) = 22.524, p<.001). Compared to this one, model 3, also including the interaction between imageability and transitivity, did not reach significance (χ^2^(2) = 0.321, p = .85).

The bayesian comparison of the full model containing the interaction against the model only comprising the main effects yielded a Bayes factor of 0.006, which also confirms the preference for the simpler model.

Regarding the direction of the main effects, highly imageable verbs were responded to significantly faster than low imageable ones. Intransitive verbs were associated to significantly longer latencies than both simple and sentential transitive verbs (ps<.001) as revealed by planned contrasts (Tukey’s HSD), but no significant differences appeared between the two transitive verb categories (p = .2).

## Discussion

In this study, verbs varying in imageability and argument structure characteristics were presented to healthy volunteers in lexical decision and grammatical decision tasks. The objective was to explore a possible greater dependence of abstract verbs on the linguistic contexts associated to them. As expected, imageability of the verbs influenced the reaction times of the participants in the two tasks. Faster responses were observed for more concrete words compared to more abstract ones. A relative disadvantage for the processing of abstract nouns has been repeatedly observed in healthy adults [1], [2]. Thus, our data replicate previous results extending their implications to the verb/action domain. The relative difficulty to process abstract words has been previously related to a greater demand on semantic retrieval and integration due to their dependence on less consistent and more diverse semantic networks [13], [16], [30].

Effects of argument structure were also observed in our data. Intransitive verbs imposed greater processing demands than transitive verbs, although no significant differences were obtained between simple and sentential transitive verbs. In our view, an enhanced capacity to process transitive, compared to intransitive, verbs could also be interpreted in terms of semantic integration demands. Lexical entries that rely on richer semantic features, and have more semantic relationships, have been argued to present lower activation thresholds than lexical items with simpler semantic content [64], [65]. In our study, the wider syntactic-semantic networks of transitive verbs would allow less integration cost and faster reaction times. On the other hand, the absence of effects of argument complexity on our data suggests that the simple or propositional nature of arguments does not affect the ease to retrieve a verb, at least during simple word-level tasks like those used in our study.

More interestingly, the interaction between imageability and argument structure characteristics did not reach significance. In the introduction, we drew the hypothesis that if the processing of abstract semantics depends more, or even exclusively, on the linguistic contexts abstract words are associated to, argument structure should differentially influence the processing of concrete and abstract verbs. The appearance of imageability and argument structure effects in our results demonstrates the sensitivity of the two tasks applied to inform lexical-semantic and grammatical-syntactic phenomena. However, the lack of a significant interaction between the two main effects shows that the complexity of the linguistic contexts associated to a word does not differentially affect the processing of concrete and abstract words. These results are in conflict with hypotheses like the dual coding theory, according to which, concrete and abstract semantics rely on verbal-dependent codes to a different extent.

In contrast, our data support a view of semantic representation that links the ease of processing to semantic richness, but does not emphasize the linguistic nature of information. In the two experiments presented here, we observed faster reaction times for verbs associated to richer contexts: concrete and transitive verbs. According to our interpretation, the processing advantage of concrete words is due to the greater availability of contextual information in memory that supports the processing of concrete semantics. Thus, a participant recognizes the transitive concrete verb “to stretch” faster than the transitive abstract verb “to promote”, not because the first presents additional sensory-based associations, but because it is more extensively linked to semantic information than the latter, in the same way that we find it easier to classify “to stretch” than the intransitive verb “to flow” because it relies on wider syntactic-semantic networks. Along with the results of previous studies [16], [30], this observation supports the relevance of context richness on semantic representation.

It should be taken into account, nevertheless, that the lack of a significant interaction between concreteness and argument structure in our study does not guarantee the independence of these two sources of information during linguistic processing. Qualitative differences between sensory-based and language-based semantic information might, hence, coexist with the influence of quantitative variations in the availability of contextual information. Some studies highlight the role of semantic richness over processing cost of concrete and abstract words, but also point out that different sources of information might contribute differentially to the processing of concrete and abstract concepts. For example, Amsel and Cree [66] presented participants with a semantic categorization task using concrete and abstract words in an event-related potential study. Their behavioural results supported the hypothesis that concrete and abstract concepts are distributed in a continuum of semantic richness. However, they found differences in the electrophysiological activity associated to the two word types, what suggests that they might be influenced by qualitatively differential sources of information. Along the same lines, Recchia and Jones [67] found differential effects of different measures of semantic richness, like number of semantic neighbours and number of semantic features, over processing cost of concrete and abstract words.

Finally, although we attempted to ensure the recruitment of grammatical-syntactic information by means of a grammatical decision task in our second experiment, the use of single-word tasks poses a limitation to the generalizability of our results to more complex and naturalistic tasks involving full sentence processing. The use of this kind of tasks in further studies might favour the appearance of qualitative differences between different classes of information.

## Supporting Information

Appendix S1 **List of experimental stimuli and percentages of appearance with the different argument structures in the syntactic corpus.**
(DOCX)

Appendix S2 **Results of the imageability survey (n = 25).**
(DOCX)

Appendix S3 **List of filler stimuli used.**
(DOCX)
