# Supplementary material for: Argument Structure and the Representation of Abstract Semantics
Source: PLoS One. 2014 Aug 11;9(8):e104645. doi: 10.1371/journal.pone.0104645 (PMC4128767; doi:10.1371/journal.pone.0104645)
Supplement: Appendix S1 — List of experimental stimuli and percentages of appearance with the different argument structures in the syntactic corpus. (DOCX) [file pone.0104645.s001.docx]

Appendix S1. List of experimental stimuli and percentages of appearance with the different argument structures in the syntactic corpus.

| **Imageability** | **Intransitive Verbs** | **Int.  %** | **Sm Tr.  %** | **St Tr.  %** | **Simple Transitive Verbs** | **Int.  %** | **Sm Tr.  %** | **St Tr.  %** | **Sentential Trastive Verbs** | **Int.  %** | **Sm Tr.  %** | **St Tr.  %** |
| --- | --- | --- | --- | --- | --- | --- | --- | --- | --- | --- | --- | --- |
| Low | adular | 100.0 | 0.0 | 0.0 | acarrear | 0.0 | 100.0 | 0.0 | aducir | 0.0 | 100.0 | 25.0 |
|  | amolar | 100.0 | 0.0 | 0.0 | acatar | 5.9 | 94.1 | 0.0 | alegar | 27.8 | 72.2 | 27.8 |
|  | cesar | 91.7 | 0.0 | 0.0 | acometer | 0.0 | 100.0 | 0.0 | asegurar | 10.4 | 84.2 | 62.1 |
|  | crepitar | 100.0 | 0.0 | 0.0 | anular | 5.3 | 94.7 | 0.0 | deducir | 9.7 | 61.3 | 51.6 |
|  | cundir | 100.0 | 0.0 | 0.0 | aplastar | 0.0 | 88.0 | 0.0 | estimar | 5.4 | 50.0 | 42.9 |
|  | decaer | 100.0 | 0.0 | 0.0 | aportar | 6.0 | 88.0 | 0.0 | impedir | 0.0 | 98.4 | 27.1 |
|  | divagar | 100.0 | 0.0 | 0.0 | captar | 0.0 | 97.4 | 0.0 | implicar | 0.0 | 88.9 | 11.1 |
|  | errar | 100.0 | 0.0 | 0.0 | causar | 0.0 | 86.7 | 0.0 | inferir | 12.5 | 75.0 | 37.5 |
|  | escasear | 100.0 | 0.0 | 0.0 | desechar | 11.1 | 88.9 | 0.0 | insinuar | 19.2 | 65.4 | 26.9 |
|  | existir | 97.9 | 0.0 | 0.0 | emitir | 9.1 | 88.6 | 0.0 | intuir | 6.7 | 93.3 | 46.7 |
|  | flaquear | 100.0 | 0.0 | 0.0 | generar | 0.0 | 92.0 | 0.0 | jurar | 7.9 | 89.5 | 51.3 |
|  | fracasar | 100.0 | 0.0 | 0.0 | invocar | 8.3 | 91.7 | 0.0 | maliciar | 0.0 | 25.0 | 50.0 |
|  | holgar | 100.0 | 0.0 | 0.0 | merecer | 0.8 | 86.3 | 3.1 | matizar | 50.0 | 50.0 | 21.4 |
|  | legislar | 100.0 | 0.0 | 0.0 | promover | 0.0 | 85.2 | 0.0 | objetar | 28.6 | 57.1 | 28.6 |
|  | malvivir | 100.0 | 0.0 | 0.0 | recobrar | 1.8 | 93.0 | 0.0 | postular | 15.4 | 84.6 | 23.1 |
|  | peligrar | 100.0 | 0.0 | 0.0 | remediar | 12.5 | 87.5 | 0.0 | precisar | 29.3 | 65.5 | 24.1 |
|  | perdurar | 100.0 | 0.0 | 0.0 | requerir | 10.0 | 88.0 | 6.0 | predecir | 12.5 | 87.5 | 37.5 |
|  | renacer | 100.0 | 0.0 | 0.0 | sortear | 0.0 | 100.0 | 0.0 | prever | 15.9 | 77.3 | 22.7 |
|  | resollar | 100.0 | 0.0 | 0.0 | superar | 9.6 | 90.4 | 0.0 | reiterar | 4.5 | 95.5 | 36.4 |
|  | variar | 88.9 | 0.0 | 0.0 | suscitar | 4.0 | 88.0 | 0.0 | sugerir | 12.3 | 80.7 | 24.6 |
| High | arder | 87.5 | 1.9 | 0.0 | acelerar | 11.1 | 88.9 | 0.0 | afirmar | 28.3 | 63.9 | 51.3 |
|  | asentir | 94.2 | 0.0 | 0.0 | afrontar | 0.0 | 100.0 | 0.0 | agregar | 46.3 | 47.8 | 28.4 |
|  | cenar | 94.0 | 0.0 | 0.0 | alumbrar | 10.0 | 90.0 | 0.0 | anunciar | 22.7 | 71.9 | 28.1 |
|  | chirriar | 100.0 | 0.0 | 0.0 | aplastar | 0.0 | 88.0 | 0.0 | avisar | 4.4 | 77.9 | 14.7 |
|  | cojear | 100.0 | 0.0 | 0.0 | apretar | 8.6 | 90.0 | 0.0 | confesar | 13.2 | 78.5 | 38.0 |
|  | crujir | 100.0 | 0.0 | 0.0 | atrapar | 0.0 | 88.5 | 0.0 | criticar | 6.3 | 91.7 | 18.8 |
|  | delirar | 100.0 | 0.0 | 0.0 | castigar | 0.0 | 100.0 | 0.0 | declarar | 27.0 | 44.7 | 38.3 |
|  | enviudar | 100.0 | 0.0 | 0.0 | devorar | 8.7 | 89.1 | 0.0 | indicar | 14.0 | 81.4 | 53.5 |
|  | estallar | 85.7 | 0.0 | 0.0 | elogiar | 7.1 | 85.7 | 0.0 | opinar | 5.0 | 29.7 | 22.8 |
|  | exagerar | 85.7 | 0.0 | 0.0 | engañar | 4.8 | 92.8 | 0.0 | ordenar | 15.0 | 60.0 | 21.0 |
|  | exclamar | 99.4 | 0.0 | 0.0 | enterrar | 11.1 | 88.9 | 0.0 | remarcar | 0.0 | 100.0 | 100.0 |
|  | florecer | 100.0 | 0.0 | 0.0 | estirar | 0.0 | 89.3 | 0.0 | rogar | 9.1 | 81.8 | 63.6 |
|  | flotar | 100.0 | 0.0 | 0.0 | filmar | 0.0 | 100.0 | 0.0 | rumorear | 0.0 | 100.0 | 100.0 |
|  | fluir | 100.0 | 0.0 | 0.0 | herir | 9.1 | 87.9 | 0.0 | señalar | 9.4 | 81.1 | 30.3 |
|  | germinar | 100.0 | 0.0 | 0.0 | limpiar | 5.8 | 87.2 | 0.0 | simular | 0.0 | 100.0 | 41.7 |
|  | recular | 100.0 | 0.0 | 0.0 | pisar | 9.3 | 90.7 | 0.0 | sostener | 9.2 | 84.5 | 20.4 |
|  | relucir | 100.0 | 0.0 | 0.0 | planchar | 0.0 | 100.0 | 0.0 | subrayar | 7.9 | 89.5 | 39.5 |
|  | trabajar | 86.5 | 14.3 | 0.0 | soltar | 7.3 | 90.1 | 0.0 | suplicar | 18.8 | 56.3 | 25.0 |
|  | trajinar | 100.0 | 0.0 | 0.0 | suprimir | 10.7 | 85.7 | 0.0 | temer | 9.5 | 73.4 | 36.7 |
|  | triunfar | 95.5 | 0.0 | 0.0 | trazar | 0.0 | 100.0 | 0.0 | tolerar | 0.0 | 100.0 | 20.7 |
