# Supplementary material for: Argument Structure and the Representation of Abstract Semantics
Source: PLoS One. 2014 Aug 11;9(8):e104645. doi: 10.1371/journal.pone.0104645 (PMC4128767; doi:10.1371/journal.pone.0104645)
Supplement: Appendix S2 — Results of the imageability survey (n = 25). (DOCX) [file pone.0104645.s002.docx]

Appendix S2. Results of the imageability survey (n=25).

|  | Mean | SD |  | Mean | SD |  | Mean | SD |  | Mean | SD |  | Mean | SD |
| --- | --- | --- | --- | --- | --- | --- | --- | --- | --- | --- | --- | --- | --- | --- |
| abrazar | 6.52 | 1.12 | danzar | 6.12 | 1.09 | incluir | 2.68 | 1.68 | predicar | 4.8 | 1.76 | sonar | 4.28 | 1.59 |
| adivinar | 4.04 | 1.90 | decaer | 2.88 | 1.56 | incurrir | 1.92 | 1.41 | proceder | 2.28 | 1.10 | sortear | 4.56 | 1.85 |
| aducir | 2.16 | 1.34 | decidir | 3.88 | 1.59 | inducir | 2.16 | 1.31 | pujar | 5.04 | 2.01 | suplicar | 5.04 | 1.77 |
| adular | 3.32 | 1.93 | decir | 4.76 | 1.64 | inferir | 1.84 | 1.14 | pulular | 3.68 | 2.14 | suplir | 2.28 | 1.28 |
| aguardar | 3.32 | 1.97 | decretar | 2.28 | 1.43 | insinuar | 2.8 | 1.63 | realizar | 3.32 | 1.86 | suponer | 1.96 | 0.98 |
| alegar | 3.24 | 1.48 | dedicar | 3 | 1.47 | jadear | 4.92 | 1.82 | rebotar | 4.84 | 1.89 | susurrar | 5.56 | 1.47 |
| aletear | 5.12 | 1.94 | delirar | 4.24 | 1.94 | lamentar | 4.4 | 1.58 | rebuscar | 3.68 | 2.06 | tañer | 3.68 | 2.36 |
| aludir | 2.4 | 1.29 | deplorar | 2.16 | 1.14 | legislar | 2.88 | 1.72 | rebuznar | 4.96 | 2.09 | tentar | 2.8 | 1.41 |
| amolar | 2.64 | 1.85 | detallar | 2.72 | 1.14 | llamear | 4.16 | 2.25 | recalcar | 3.24 | 1.90 | tiritar | 5.8 | 1.61 |
| anidar | 5 | 1.80 | detonar | 4.72 | 2.17 | magrear | 4.92 | 2.08 | rechinar | 4.04 | 2.03 | titilar | 3.32 | 2.04 |
| apreciar | 2.68 | 1.46 | divagar | 2.48 | 1.33 | maliciar | 2.2 | 1.08 | reciclar | 5.08 | 2.00 | tolerar | 2 | 1.00 |
| apuntar | 6.24 | 1.23 | dormitar | 4.76 | 2.09 | malvivir | 2.64 | 1.32 | recular | 4.28 | 1.88 | toser | 6.44 | 1.08 |
| arar | 6.04 | 1.24 | empezar | 2.8 | 1.47 | mantener | 2.8 | 1.83 | referir | 2.36 | 1.55 | trajinar | 4.08 | 1.89 |
| atracar | 5.92 | 1.50 | enviudar | 4.28 | 1.97 | matizar | 2.08 | 1.08 | refluir | 2.44 | 1.64 | trincar | 3.24 | 1.94 |
| bascular | 4.4 | 2.14 | errar | 2.84 | 1.28 | mendigar | 5.68 | 1.57 | reiterar | 3.12 | 1.67 | trotar | 6.04 | 1.27 |
| berrear | 4.72 | 2.01 | escasear | 2.88 | 1.74 | merendar | 6.2 | 0.91 | relatar | 4.36 | 1.91 | usar | 4.24 | 1.59 |
| bostezar | 6.12 | 1.39 | estimar | 2.76 | 1.56 | molar | 2.88 | 1.62 | remarcar | 3.36 | 1.55 | utilizar | 4.6 | 1.66 |
| bramar | 4.44 | 2.04 | estorbar | 3.6 | 1.83 | moquear | 5.16 | 1.99 | replicar | 3.2 | 1.61 | valorar | 2.48 | 1.26 |
| brincar | 5.8 | 1.35 | exclamar | 4.24 | 1.64 | mutilar | 4.84 | 2.08 | reponer | 4.2 | 2.02 | vibrar | 4.64 | 1.98 |
| bucear | 6.4 | 0.76 | faenar | 3.52 | 1.73 | objetar | 2.12 | 1.20 | reptar | 5.04 | 1.90 | vocear | 4.8 | 1.94 |
| captar | 2.44 | 1.08 | fallecer | 5.24 | 1.94 | otorgar | 3.36 | 2.08 | requerir | 1.76 | 0.97 | zozobrar | 3.44 | 1.98 |
| censurar | 3.56 | 2.02 | figurar | 2.72 | 1.65 | pastar | 5.08 | 1.71 | resollar | 2.76 | 1.51 |  |  |  |
| chillar | 6.12 | 0.97 | flaquear | 3.12 | 1.67 | patalear | 5.88 | 1.56 | resonar | 3.36 | 1.68 |  |  |  |
| chirriar | 4.2 | 1.98 | fornicar | 6.24 | 1.13 | patinar | 6.68 | 0.63 | retrucar | 2.44 | 1.83 |  |  |  |
| clarear | 3.56 | 1.78 | fracasar | 2.64 | 1.25 | pedalear | 5.88 | 1.51 | retumbar | 3.44 | 1.83 |  |  |  |
| cojear | 6 | 1.58 | galopar | 5.88 | 1.51 | peligrar | 3.2 | 2.00 | rogar | 5.04 | 1.49 |  |  |  |
| comer | 6.6 | 1.04 | gemir | 5.04 | 1.79 | perdurar | 2.2 | 1.35 | roncar | 5.92 | 1.50 |  |  |  |
| concluir | 3.16 | 1.60 | germinar | 4.52 | 2.14 | perecer | 3.8 | 2.12 | rotar | 5.28 | 1.54 |  |  |  |
| convenir | 2.32 | 1.52 | gimotear | 4.84 | 1.93 | piar | 5.2 | 1.66 | rugir | 6.2 | 1.19 |  |  |  |
| crepitar | 3.28 | 2.05 | graznar | 4.6 | 1.96 | pitar | 5.72 | 1.31 | rumorear | 3.84 | 1.89 |  |  |  |
| croar | 5.48 | 1.64 | holgar | 2.52 | 1.61 | platicar | 5.24 | 1.92 | saltar | 6.68 | 0.63 |  |  |  |
| crujir | 4.72 | 1.74 | husmear | 4.24 | 1.90 | podar | 5.8 | 1.38 | sentir | 2.84 | 1.77 |  |  |  |
| cundir | 2.2 | 1.08 | implicar | 2.4 | 1.22 | postular | 2.04 | 1.02 | sollozar | 5.24 | 1.71 |  |  |  |
