# Supplementary material for: Argument Structure and the Representation of Abstract Semantics
Source: PLoS One. 2014 Aug 11;9(8):e104645. doi: 10.1371/journal.pone.0104645 (PMC4128767; doi:10.1371/journal.pone.0104645)
Supplement: Appendix S3 — List of filler stimuli used. (DOCX) [file pone.0104645.s003.docx]

Appendix S3. List of filler stimuli used.

| List of pseudo-verb stimuli used in experiment 1 | | | |  |  |
| --- | --- | --- | --- | --- | --- |
| acipar | asopar | custir | eromir | iudigerar | resicir |
| acapar | avectar | daliar | escazar | liltiar | resiscar |
| aconcir | avinar | daltear | esiner | maldatir | rultar |
| aconter | avuser | decedir | etnastir | mesar | runtular |
| acupar | bimisear | decorrar | excrigar | necenar | sececir |
| adacar | bomar | dereir | fegerlar | notenar | seconar |
| afelgar | brejer | derotrar | fernanar | ocarar | sequisar |
| allicar | buecerar | derucer | fledar | paraciar | seser |
| allietar | calcesar | derusar | flunir | pasfurar | sufragir |
| allipar | cascazar | devuser | fuzmar | patamar | sullair |
| allistar | cecoar | dezuerer | gelir | peratrar | sullasar |
| alondrar | chajar | dicusar | gulgar | plebatar | sumpatar |
| amabunar | cinar | digizar | ibrar | plubir | suntenir |
| amagorar | cisar | drantrar | imnerer | pravanar | travimar |
| amaproer | clacicar | duverer | imnolar | preracar | trirreir |
| amiboter | clafioar | empetrar | incasar | preveser | tristmar |
| ancer | clicacar | encarrar | indiar | prosopir | tucenar |
| anmabear | clocecir | enroldar | insanir | recetrar | unenar |
| aplondar | coltar | ensibar | irceder | rellirar | utetar |
| ascoldar | cuilar | envinar | isfracar | reratar | zunar |

| List of non-verb stimuli used in experiment 2 | | | |  |  |
| --- | --- | --- | --- | --- | --- |
| ajuar | cadáver | ejemplar | irregular | olivar | secular |
| alcázar | calamar | elixir | láser | orbicular | seglar |
| alfiler | cáncer | escolar | lenticular | paladar | similar |
| almíbar | canciller | espectacular | líder | palomar | singular |
| alquiler | capilar | estelar | lucifer | papilar | solar |
| altar | carácter | éter | lumbar | particular | souvenir |
| ámbar | caviar | familiar | lunar | peculiar | suéter |
| angular | celular | fular | manjar | peninsular | sumiller |
| anteayer | chófer | ganglionar | mártir | pilar | taller |
| auricular | coclear | gángster | maxilar | pinar | telar |
| avatar | collar | géiser | medular | placer | tercer |
| azúcar | corpuscular | glaciar | melonar | polar | tubular |
| bachiller | cráter | glandular | menester | porvenir | ultramar |
| bajamar | crepuscular | globular | mercader | póster | unicelular |
| bazar | cuadrangular | hámster | militar | postrer | valvular |
| bereber | dactilar | hazmerreír | molecular | preliminar | vascular |
| billar | dispar | húsar | nácar | prócer | váter |
| bipolar | dólar | impar | neceser | púber | visir |
| brigadier | doquier | insular | néctar | quehacer | vulgar |
| bulevar | dossier | intracelular | ocular | rectangular | yugular |
